# Maternal per- and poly-fluoroalkyl substances exposures associated with higher depressive symptom scores among immigrant women in the Chemicals in Our Bodies cohort in San Francisco

**DOI:** 10.1016/j.envint.2023.107758

**Published:** 2023-01-16

**Authors:** Max T. Aung, Stephanie M. Eick, Amy M. Padula, Sabrina Smith, June-Soo Park, Erin DeMicco, Tracey J. Woodruff, Rachel Morello-Frosch

**Affiliations:** a Department of Population and Public Health Sciences, Keck School of Medicine, University of Southern California, Los Angeles, CA, USA; b Gangarosa Department of Environmental Health, Rollins School of Public Health, Emory University, Atlanta, GA, USA; c Program on Reproductive Health and the Environment, Department of Obstetrics, Gynecology and Reproductive Sciences, University of California, San Francisco, San Francisco, CA, USA; d Environmental Chemistry Laboratory, Department of Toxic Substances Control, California Environmental Protection Agency, Berkeley, CA, USA; e Department of Environmental Science, Policy and Management and School of Public Health, University of California, Berkeley, Berkeley, CA, USA

**Keywords:** PFAS, Maternal, Depressive symptoms, Mixtures, Immigrants

## Abstract

**Background:**

Exposure to per- and poly-fluoroalkyl substances (PFAS) remains an important public health issue due to widespread detection and persistence in environmental media, slow metabolism in humans, and influences on physiological processes such as neurological signaling. Maternal depression is highly prevalent during pregnancy and postpartum and is potentially sensitive to PFAS. The health risks associated with PFAS may be further amplified in historically marginalized communities, including immigrants.

**Objective:**

Evaluate maternal concentrations of PFAS in association with depression scores during pregnancy and whether effects differ between US born and immigrant women.

**Methods:**

Our study sample included 282 US born and 235 immigrant pregnant women enrolled in the Chemicals in Our Bodies prospective birth cohort based in San Francisco, CA. We measured 12 PFAS in serum samples collected in the second trimester and depressive symptom scores were assessed using the Center for Epidemiologic Studies Depression Scale. Associations were estimated using linear regression, adjusting for maternal age, education, pre-pregnancy body mass index, and parity. Associations with a PFAS mixture were estimated using quantile g-computation.

**Results:**

In adjusted linear regression models, a twofold increase in two PFAS was associated with higher depression scores in the overall sample, and this association persisted only among immigrant women (*β* [95 % confidence interval]: perfluorooctane sulfonic acid (2.7 [0.7–4.7]) and methyl-perfluorooctane sulfonamide acetic acid (2.9 [1.2–4.7]). Quantile g-computation indicated that simultaneously increasing all PFAS in the mixture by one quartile was associated with increased depressive symptoms among immigrant women (mean change per quartile increase = 1.12 [0.002, 2.3]), and associations were stronger compared to US born women (mean change per quartile increase = 0.09 [-1.0, 0.8]).

**Conclusions:**

Findings provide new evidence that PFAS are associated with higher depression symptoms among immigrant women during pregnancy. Results can inform efforts to address environmental factors that may affect depression among US immigrants.

## 1. Introduction

Endocrine disrupting chemicals such as per- and poly-fluoroalkyl substances (PFAS) are major environmental contaminants of health concern due to their increasing ubiquity and persistence in environmental media, including drinking water (Sunderland et al., 2019). Biomonitoring studies indicate that widespread environmental contamination from consumer products and historic manufacturing has led to detection of multiple PFAS in nearly all humans and wildlife (Calafat et al., 2007; De Silva et al., 2021; Sunderland et al., 2019). While regulations and phaseouts have led to reductions of some PFAS in humans (Hurley et al., 2018), given the long half-life of many of these compounds, there is a need to concurrently determine health risks associated with multiple PFAS exposures to better inform human health risk assessments.

There is increasing evidence that human exposure to PFAS affects several intermediate biological mechanisms, including inflammation, oxidative stress, hormone signaling, and lipid metabolism (Fenton et al., 2021). While PFAS can accumulate in various tissues in the human body, a recent review highlighted evidence from human, animal, and *in vitro* studies indicating that PFAS can accumulate in the brain and nervous tissue potentially by interfering with tight junctions at the blood brain barrier and interacting with transmembrane transporters (Cao and Ng, 2021). Importantly, PFAS may elicit neurotoxic effects through various mechanisms, including disrupting calcium dependent signaling and interfering with neurotransmitter signaling (Cao and Ng, 2021).

Perinatal mood and anxiety disorders occur among 1 in 7 women in the United States (US) during pregnancy and in the postpartum period (Dagher et al., 2021). Pre- and perinatal depression has downstream implications for adverse outcomes, including increased risk for maternal and perinatal morbidity (e.g., preterm birth, postnatal depression) (Dagher et al., 2021). The economic costs of mood and anxiety disorders during pregnancy and postpartum, including depression, was estimated to be $14.2 billion in 2017 (Luca et al., 2019). Both social and environmental factors can influence the onset and severity of depression (Mutic et al., 2021). For example, structural racism and perceived discrimination may contribute to disparities in depression rates and severity (Hankerson et al., 2022). Furthermore, immigration status may intersect with structural racism and contribute to higher depression rates among immigrant women (Alegría et al., 2017; O’Mahony et al., 2013; Snow et al., 2021). Chronic stressors attributable to social factors have been shown in animal models to influence neurological health through interfering with the hypothalamic-pituitary–gonadal and adrenal axes and stress hormone signaling, such as glucocorticoids (Herzog et al., 2009; McCormick and Green, 2013). Given that PFAS can act as endocrine disruptors, the dual exposure of social stressors and PFAS may enhance the risk of neurobehavioral conditions. Thus, it is critical to advance understanding of risk factors of depression with close attention towards marginalized immigrant communities.

In addition to understanding social factors that influence perinatal depression disparities, there is a need to determine the contribution of potential environmental neurotoxicants such PFAS. Despite evidence of the toxic effects of PFAS, no studies have assessed the relationship between PFAS and prenatal depressive symptoms. To address this knowledge gap, we assessed relationships between prenatal PFAS exposures and maternal depressive symptoms during pregnancy. Furthermore, we sought to determine the extent to which these associations differed between US born and immigrant women. We hypothesized that PFAS exposure increases the risk of prenatal depression, and that this relationship is amplified among immigrant women partly due to structural factors and social stressors that are unique to immigrants.

## 2. Methods

### 2.1. Study population

Participants in this study are part of the Chemicals in Our Bodies (CIOB) prospective pregnancy cohort based in San Francisco, CA. A detailed description of the study population is provided elsewhere (Eick et al., 2021, 2020). Briefly, recruitment of study participants occurred during the second trimester of pregnancy at three University of California, San Francisco (UCSF) hospitals. The inclusion and eligibility criteria for participation included being at least 18 years of age, able to speak English or Spanish as a primary language and having a singleton pregnancy. Immigrant status was assessed through a survey question asking about the study participant’s country of birth. All study participants provided written, informed consent prior to participation, and the Institutional Review Boards at UCSF (13–12160) and the University of California, Berkeley (2010–05–04) approved the CIOB study.

### 2.2. PFAS exposure assessment

Serum was collected during the second trimester (range of 12–28 weeks’ gestation) and were stored at −80 °C. Serum samples were analyzed for PFAS at the Environmental Chemical Laboratory at the California Department of Toxic Substances Control (DTSC). Samples were injected into an automated on-line solid phase extraction system coupled to liquid chromatography and tandem mass spectrometry using methods previously described in detail (Eick et al., 2021, 2020). Twelve unique PFAS compounds were measured: perfluorobutane sulfonate (PFBS), perfluorohexanesulphonic acid (PFHxS), perfluorooctane sulfonic acid (PFOS), perfluoroheptanoic acid (PFHpA), perfluorooctanoic acid (PFOA), perfluorononanoic acid (PFNA), perfluorodecanoic acid (PFDeA), perfluoroundecanoic acid (PFUdA), perfluorododecanoic acid (PFDoA), perfluorooxtane sulfonamide (PFOSA), methyl-perfluorooxtane sulfonamide acetic acid (Me-PFOSA-AcOH), and ethyl-perfluorooctane sulfonamide acetic acid (Et-PFOSA-AcOH). Among these quantified compounds, we focused downstream analyses on those with at least 70 % detection rates, which led to a final PFAS roster of PFOA, PFOS, PFNA, PFHxS, PFDeA, PFUdA, and Me-PFOSA-AcOH. This cutpoint was chosen for consistency with our prior work in this study population (Eick et al., 2022a; Taibl et al., 2022). For any values that were below the method detection limit (MDL), we imputed the machine read value if it was available. We chose this imputation method in order to maximize all available data and preserve a more realistic exposure distribution, as setting all minimally detected values to the same value in reality is implausible (Hornung and Reed, 1990).

### 2.3. Depression symptom score assessment

During the second trimester, we administered the Center for Epidemiological Studies-Depression (CES-D), a validated, self-report instrument, to assess depressive symptoms. The CES-D collects participants’ responses to a 20-item questionnaire to measure symptoms of depression that have occurred in the prior year, which allows for assessment of longer-term depressive symptoms leading up to pregnancy. The questionnaire includes factors such as feeling unusually bothered, having poor appetite, feeling hopelessness, trouble focusing, feeling fearful, trouble sleeping, feeling lonely, pessimism, and fatigue (Radloff, 1977). The CES-D has been evaluated for its measurement properties and appropriateness for application toward assessing depressive symptoms in diverse samples of pregnant women (Canady et al., 2009). CES-D scores can range from 0 to 60. While the total CES-D score can be set to a binary value at or above 16 to identify those at risk of depression (Radloff, 1977), we selected to use this binary categorization only for descriptive statistics and modeled the continuous CES-D score for all regression models. The rationale for this was that modeling continuous CES-D score allows for estimation of the relative increase in depressive symptoms and is sufficiently powered for models with limited sample size.

### 2.4. Statistical approach

We estimated univariate distributions of PFAS chemicals in our overall sample and stratified by immigrant status and estimated Spearman correlation coefficients between compounds. We also estimated PFAS concentrations and tabulated demographic and social factors between participants with likely depression and no depression (≥16 vs < 16). When we tested for bivariate associations, we transformed each PFAS using the natural log to obtain normal distributions and conducted t-tests and chi-squared tests for continuous and categorical variables, respectively.

For single pollutant analyses, we built multiple linear regression models with CES-D scores as a continuous outcome and individual natural log transformed PFAS metabolites as independent exposures. For adjusted models, we considered potential confounders and determined final covariates based on bivariate associations (e.g., covariates independently associated with both PFAS and CES-D scores), which included: maternal age, education, pre-pregnancy body mass index (BMI), parity, and immigrant status. Other factors that influenced final model selection included missingness of covariates. We specified models with other social hardship measures (e.g., financial strain, food insecurity) and found that they did not appreciably change the magnitude or direction of effect estimates and reduced statistical power. To identify evidence of effect modification by immigrant status, we modified adjusted models to include an interaction term between individual PFAS and immigrant status. While single time point BMI or change in BMI during pregnancy may be a mediator for PFAS and CES-D score measures during pregnancy, we conceptualized pre-pregnancy BMI to serve as a proxy for dietary and physical behavior characteristics prior to pregnancy, which would be temporarily and biologically consistent with a confounder for PFAS and CES-D scores measured during pregnancy. We then stratified our sample based on immigrant status, therefore including all covariates except immigrant status. We observed evidence of non-linearity in CES-D scores, and we explored transformations [ln (CES-D + 1)] as potential sensitivity analyses, and based on comparable residual distributions and greater interpretability, we modeled CES-D scores without transformation in final models. Also, for interpretability, we converted final single pollutant regression results to indicate the association between a twofold (100 %) increase in a given PFAS associated with a change in CES-D scores. As a sensitivity analysis, we evaluated potential exposure–response relationships between the most robust associations for PFAS and CES-D scores by categorizing the exposure into quartiles.

For mixtures analyses, we applied quantile g-computation, which estimates the cumulative effect of simultaneously increasing all PFAS by one quartile within the mixture using a parametric, generalized linear based implementation of g-computation (Keil et al., 2020). With this approach, individual PFAS are allowed to have opposing directions of effect relative to CES-D scores, which are translated to negative and positive weights that sum to 1 and are indicative of relative compound importance either in the negative or positive directions.

## 3. Results

Table 1 reports key covariates relative to a CES-D score cut-off of 16 for individuals with symptoms indicating risk for clinical depression (N = 42 participants [8.1 %]). The CIOB study sample was comprised largely of White (42.2 %) and Latina (32.4 %) participants. Approximately 56 % of participants were US born and 44 % were immigrants, and most of the immigrant participants were Latina (62 %) followed by Asian American/Pacific Islander (22 %). Half of the study participants reported that their current pregnancy was their first. Most participants (65.2 %) had college-level educational attainment and reported having no history of smoking (95.3 %). Each of these covariates were associated with risk of depression (Table 1). For example, risk of depression was highest among Latina participants (66.7 %), women who did not graduate college (82.5 %), and immigrant participants (63.2 %).

The crude distributions of two PFAS compounds differed by depression status (Table 2). PFOS and PFUdA were both lower in those with risk of depression ([PFOS_Med_] = 1.6 ng/mL; [PFUdA_Med_] = 0.06 ng/mL) compared to those without risk of depression ([PFOS_Med_] = 2.0 ng/mL; [PFUdA_Med_] = 0.1 ng/mL). PFAS compounds were all positively correlated, and we observed the smallest positive correlation between PFUdA and MePFOS (ρ = 0.1) and the largest positive correlation between PFDeA and PFNA (ρ = 0.7) (Supplemental Table 1). Stratification by immigrant status also indicated that all PFAS levels were lower among immigrants compared to US born participants. The greatest difference was observed for PFOS, where US born women had a median of 2.2 ng/mL, while immigrant women had a median of 1.5 ng/mL.

We observed associations between single pollutants and PFAS mixtures in relation to depression scores (Table 3). In the overall sample, we observed that a twofold (100 %) increase in PFOS was associated with 1.5 unit increase in CES-D scores (95 % confidence interval [CI] = 0.2, 2.9) and Me-PFOSA-AcOH was associated with a 1.4 unit increase in CES-D scores (95 % CI = 0.5, 2.4). In adjusted models with interaction terms between PFAS and immigrant status, Me-PFOSA-AcOH significantly interacted with immigrant status (p < 0.05). When stratifying by immigrant status, we observed that these associations were largely driven and more amplified among immigrant compared to US born women. A twofold (100 %) unit increase in PFOS in immigrant women was associated with a 2.7 unit increase in CES-D scores (95 % CI = 0.7, 4.7), while Me-PFOSA-AcOH was associated with a 2.9 unit increase in CES-D score (95 % CI = 1.2, 4.7). Evaluation of a potential dose–response for Me-PFOSA-AcOH did not indicate a clear exposure–response relationship. For example, in the overall sample, the second quartile of Me-PFOSA-AcOH was not significantly higher than the reference (first quartile), however the third quartile was associated with 2.3 unit higher CES-D score (95 % CI = 1.0, 3.3.6) and the fourth quartile was associated with 1.9 unit higher CES-D score (95 % CI = 0.6, 3.3) (Supplemental Table 2).

Quantile g-computation indicated similar trends, where simultaneously increasing all PFAS in the mixture by one quartile was associated with a greater increase in CES-D scores in immigrant women (mean change per quartile increase = 1.1, 95 % CI = 0.002, 2.3) compared to US born women (mean change per quartile increase = −0.09, 95 % CI = −1.0, 0.8). Decomposition of quantile g-computation effect estimates indicated that Me-PFOSA-AcOH and PFOS exhibited the greatest positive weights to the overall effect relative to the other PFAS compounds in the overall sample (Supplemental Table 3). In stratified quantile g-computation models there were more positive weights from individual PFAS in immigrant women (Me-PFOSA-AcOH, PFDeA, PFOS, PFHxS, PFNA) compared to US born women (Me-PFOSA-AcOH, PFOS, PFDeA), although Me-PFOSA-AcOH remained a top contributor to the overall mixture in both strata (Supplemental Table 3).

## 4. Discussion

In this study we investigated the relationship between maternal prenatal CES-D scores in the CIOB cohort and prenatal serum PFAS concentrations. Importantly we investigated stratified associations based on immigrant status to better understand potential effect modification by this important social identity. Our analysis provides evidence that two long-chained PFAS compounds, PFOS and Me-PFOSA-AcOH, are associated with higher pre-pregnancy depressive symptom scores, and that this association was more pronounced among immigrant participants. Formal tests for interaction revealed evidence of effect modification with Me-PFOSA-AcOH.

Comparison of mixtures analysis results and linear regression results revealed some differences between the two modeling approaches. Our mixtures analysis suggests that simultaneous exposure to multiple PFAS was associated with increased CES-D scores. As with single pollutant models, the associations between the overall PFAS mixture and higher depression scores were evident largely among immigrant women and not US born women. However, individual contributions of PFAS compounds in the quantile g-computation model differed between US born and immigrant stratified mixtures analyses. It is possible that these differences are due to differences in exposure sources and patterns (Environmental Protection Agency, 2000; Rickard et al., 2022), as PFAS such as PFUdA are longer chained *(i.e., more carbon molecules)* relative to PFOA and PFOS, and different technical mixtures of PFAS have been used in specialized applications, and we observed that levels of these PFAS were lower among immigrant women in our study. Additionally, while there may be differences in relative quantile g-computation weights of individual PFAS among immigrant versus US born women, the overall effect estimate for the PFAS mixture for US born women is close to null.

One other study conducted in 2013 has investigated PFAS exposures among immigrants, and this was focused on potential exposures through consumption of fish among refugees and immigrants from Myanmar who resided in Buffalo NY (Liu et al., 2022). Interestingly, the study found that immigrants from Myanmar had substantially higher levels of several PFAS, including PFOS (median = 35.6 ng/mL) compared to a sample of licensed anglers (median = 11.6 ng/mL) and the general NHANES population from 2013 to 2014 (5.6 ng/mL). In contrast, our more recent study found PFAS was lower in all immigrant women compared to US born women, which may partly be driven by historical trends in decreasing PFAS levels due to phase-outs and remediation efforts. The stronger associations we observed between PFOS and Me-PFOSA-AcOH and higher depressive symptom scores among immigrant women suggest that this may be a vulnerable population of concern. Immigrants can experience marginalization, which in turn can influence their exposures to social stressors such as discrimination, social isolation, acculturative stress, financial hardship, and barriers to healthcare and educational attainment (Gee et al., 2016; Miranda et al., 2005; Zelkowitz et al., 2008). Co-exposure to social stressors alongside PFAS exposures may enhance or amplify depressive symptoms even when there are lower average levels of PFAS in immigrants compared to US born persons. Animal models have independently shown effects of PFAS on neurological outcomes (e.g. neuroendocrine disruption, motor activity, stress response) (Piekarski et al., 2020), as well as effects of social stress on neurological outcomes (Blanchard et al., 2001). While joint exposures of PFAS and social stressors are lacking in animal models, some parallels can be drawn from previous studies of joint exposures to social stress and other xenobiotic exposures (e.g. drugs), with evidence of adverse neurobehavioral outcomes (e.g. increased drug abuse traits) associated with both increased social stressors and drug intake doses (Neisewander et al., 2012). Additionally, while not directly applicable to neurobehavioral outcomes, previous human studies have found that PFAS and psychosocial stressors jointly increase risk of reduced fetal growth (Eick et al., 2023), and altered maternal corticotrophin-releasing hormone levels involved in the stress response (Eick et al., 2022b). Thus, greater attention needs to be given to joint PFAS and social stress exposure–response functions for health outcomes in marginalized groups, even when their exposure levels may be lower than national averages.

One previous study (N = 377) has investigated prenatal PFAS, polybrominated diphenyl ethers (PBDE) and postpartum maternal depressive symptoms (Beck Depression Inventory-II [BDI-II]) in the Health Outcomes and Measures of the Environment (HOME) study (Vuong et al., 2020). In this study, PBDEs were associated with increased BDI-II scores, but PFAS were not associated with BDI-II postpartum. While the timing of depressive symptom assessment was different from our study, and the proportion of immigrant participants is unclear, if participants in the HOME study were largely US born, then PFAS findings would align with what we observed among US born participants in our study (Vuong et al., 2020). There are also important parallels that can be drawn from studies that have focused on neurobehavioral development in children, since this may be an antecedent to psychiatric conditions later in life. For example, the Health Outcomes and Measures of the Environment (HOME) study investigated prenatal and childhood PFAS exposures at ages 3 (n = 146) and 8 (n = 193) in association with parental-report responses using the Behavioral Assessment System for Children-2 (BASC-2) (Vuong et al., 2021). Although childhood PFAS exposures were not found to be associated with neurobehavioral features in this study, prenatal PFOS exposure was associated with higher BASC-2 scores for externalizing problems, hyperactivity, aggression and conduct problems (Vuong et al., 2021). A separate study based in the Faroe Islands, found prenatal maternal (n = 449) PFOS to be modestly associated with lower childhood cognitive scores using the Boston Naming Test (Oulhote et al., 2019). A previous review has also highlighted that overall there are mixed findings, and noted some where null associations were observed between PFAS compounds and some childhood neurobehavioral outcomes such as attention deficit hyperactivity disorder (Liew et al., 2018). A *meta*-analysis identified evidence that childhood externalizing problems were associated with subsequent adult depression risk (Loth et al., 2014). While direct comparisons from these studies cannot be made with findings from our present study, developmental effects observed in these studies provide biological plausibility that PFAS can affect neurological health and thus potentially adult depression risk.

Neuroinflammation and subsequent impacts on neuronal signaling have been hypothesized as potential precursors in the etiology of depression (Troubat et al., 2021). The potential mechanisms of action linking PFOS and Me-PFOSA-AcOH to neurological outcomes may be driven by neuroinflammation, intracellular calcium levels in affected neurons, and alterations with neurotransmitters (Cao and Ng, 2021). For example, a study of pregnant women (n = 725) based in Shanghai, China, determined that prenatal PFAS mixtures was associated elevated brain derived neurotrophic factor (BDNF) in cord blood of male offspring (Yu et al., 2021). BDNF is a peptide largely expressed in the central nervous system that is involved with neuronal development and plasticity, hence elevated cord blood levels may be indicative of neurotoxic effects induced by PFAS (Hao et al., 2017; Yu et al., 2021). Relatedly, animal models have shown evidence that PFOS and PFAS mixtures can affect neurotransmitter concentrations, such as acetylcholine and cholinergic system signaling (Cao and Ng, 2021; Foguth et al., 2020; Johansson et al., 2008). Evidence from animal models also indicates that long-chained PFAS (e.g., PFOS and Me-PFOSA-AcOH) may cross the blood brain barrier with slightly more efficiency than shorter chained PFAS, and a recent human study indicates that PFAS detection in cerebral spinal fluid correlates with elevated c-reactive protein, indicating a coupled inflammatory relationship with PFAS crossing the blood brain barrier (Cao and Ng, 2021; Dassuncao et al., 2019; Wang et al., 2018). Altogether, these potential mechanisms should be explored in future studies of PFAS and depression to contextualize intermediate physiological states attributable to the associations we observed in our study.

Given that PFAS are endocrine disrupting chemicals, their influence on neurological health may also be driven through hormone disruption. For example, a previous cross-sectional study in NHANES (n = 1,886) showed that several PFAS, including PFOS, were associated with higher total and free testosterone, in addition to non-linear associations with sex hormone binding globulin (Xie et al., 2021). Another prospective birth cohort study in Shanghai (n = 1,842) reported positive associations between PFAS mixtures and the thyroid hormone free thyroxine as well as a non-linear relationship between PFOS and thyroid stimulating hormone (Aimuzi et al., 2020). Hormone disruption may be an important factor linking PFAS exposures to depression outcomes due to the influence of hormones on neuroinflammation (Slavich and Sacher, 2019), and this relationship should be explored in future studies to contextualize our findings.

Our study had several limitations. First, our indicator of depression was based on self-report and we do not have a clinical confirmed depression outcome measure; however, the CES-D is validated and has been widely used during pregnancy (Heller et al., 2022). Second, while our study design was cross-sectional (serum samples and the CES-D were collected at the same second trimester visit), CES-D scores evaluate depressive symptoms in the last year which raises potential temporality issues related to PFAS exposures and depressive symptom outcomes. Given the long half-life of PFAS, it is unlikely that our results would be subject to reverse causality. However, the assessment of exposure after timing of the outcome underscores that we assume that current PFAS concentrations are a proxy of previous PFAS concentrations in the last year. Thus, there is potential exposure misclassification in the exposure–response function estimated through our regression models, and future studies should build on this work with a prospective study design. In addition, we did not measure usage of anti-depressants, social support, and social stress, as well as other co-morbidities of relevance for depression during pregnancy such as anxiety. Future studies should incorporate these variables and more comprehensively assess depression and potential co-morbidities such as anxiety during pregnancy as well as during the post-partum period. Another limitation of our study is the absence and insufficient power for data disaggregation among different groups of immigrants. This is an important consideration because evidence from a recent study leveraging the National Health Interview Study indicated that among immigrants, the highest rates of depression were observed among immigrants from Mexico, Central America, or the Caribbean (Flores Morales and Nkimbeng, 2021). We are unable to determine if there are specific immigrant ethnic identities that may experience greater susceptibility to PFAS related depression symptom risk. Nonetheless, our cohort had substantially balanced and high numbers of immigrant women to assess risk of depressive symptoms. Future studies should expand data disaggregation efforts. Despite these limitations, our study contained notable strengths. The CiOB is a well-characterized cohort and is highly diverse in terms of self-identified race/ethnicity, immigration status, and socioeconomic status. The CiOB cohort has relatively high proportions of Latina and Asian American immigrants, two of the fastest growing immigrant populations in the US (Abby Budiman et al., 2020). Finally, our application of quantile g-computation to assess PFAS mixtures advances greater understanding of cumulative PFAS in association with depressive symptoms.

In conclusion, results from our study find that prenatal PFAS exposure is a significant risk factor for depressive symptoms among immigrant women during pregnancy. These findings have important implications for contextualizing unique environmental risk factors among immigrants and can help inform regulatory and health protective policies surrounding immigrants and mental health. Future studies should confirm these findings among other immigrant groups, more comprehensively assess depressive and anxiety symptoms during the prenatal and postpartum periods and elucidate intermediate effect biomarkers and mechanistic pathways for understanding the link between PFAS and depressive symptoms in this uniquely vulnerable population.

## Supplementary Material

1

## Figures and Tables

**Table 1:** Chemicals in Our Bodies cohort descriptive statistics by depression indicator.

	Not Depressed	Risk of Depression	P-value
Population Characteristics	N = 479	N = 42	
Maternal Age [mean (SD)]	33.6 (5.2)	30.7 (5.9)	*<*0.01
BMI [mean (SD)]	25.4 (5.4)	26.9 (4.9)	0.07
	Count (Percent)		
Parity			*<*0.01
One Pregnancy	247 (52.8 %)	11 (27.5 %)	
Multiple Pregnancies	221 (47.2 %)	29 (72.5 %)	
Maternal Race/Ethnicity			*<*0.01
Non-Hispanic White	213 (45.0 %)	*<*5	
Non-Hispanic Black	24 (5.1 %)	*<*5	
Non-Hispanic Asian + PI + Native	82 (17.3 %)	6 (15.4 %)	
Latina/Hispanic	140 (29.6 %)	26 (66.7 %)	
Non-Hispanic Multiracial	14 (3.0 %)	*<*5	
Maternal Education			*<*0.01
Less than High School	40 (8.4 %)	12 (30.0 %)	
High School + some college	106 (22.4 %)	21 (52.5 %)	
College degree	120 (25.3 %)	*<*5	
Graduate degree	208 (43.9 %)	*<*5	
Immigrant Status			0.02
US born	250 (57.7 %)	14 (36.8 %)	
Immigrants	183 (42.3 %)	24 (63.2 %)	
Smoking status			0.09
Never	431 (95.8 %)	36 (90.0 %)	
Ever smoker	10 (2.2 %)	*<*5	
Current smoker	9 (2.0 %)	*<*5	
Financial Strain			*<*0.01
No	269 (68.3 %)	*<*5	
Yes	125 (31.7 %)	28 (90.3 %)	

**Table 2:** PFAS distributions in relation to depression risk and immigrant status.

		Not Depressed (CES-D score *<* 16)	Risk of depression (CES-D score ≥ 16)		US born	Immigrants	
PFAS compounds (ng/mL) [median (25th and 75th percentile)]	MDL^1^	N = 479	N = 42	P-value	N = 282	N = 235	P-value
PFNA	[0.04, 0.06]	0.3 [0.2; 0.4]	0.3 [0.2; 0.4]	0.19	0.3 [0.2; 0.5]	0.2 [0.2; 0.4]	*<*0.01
PFOA	[0.06, 0.13]	0.8 [0.5; 1.2]	0.7 [0.5; 0.9]	0.21	0.9 [0.6; 1.3]	0.6 [0.4; 0.9]	*<*0.01
PFHxS	[0.01, 0.02]	0.3 [0.2; 0.6]	0.3 [0.2; 0.4]	0.02	0.4 [0.3; 0.7]	0.2 [0.2; 0.4]	*<*0.01
PFOS	[0.06, 0.07]	2.0 [1.3; 3.1]	1.6 [1.05; 2.6]	0.15	2.2 [1.5; 3.3]	1.5 [1.0; 2.7]	*<*0.01
Me-PFOSA-AcOH	[0.01]	0.04 [0.03; 0.07]	0.05 [0.04; 0.08]	0.04	0.05 [0.03; 0.08]	0.04 [0.02; 0.05]	*<*0.01
PFDeA	[0.06, 0.08]	0.1 [0.08; 0.2]	0.08 [0.06; 0.2]	0.03	0.1 [0.08; 0.2]	0.1 [0.07; 0.2]	0.09
PFUdA	[0.03, 0.05]	0.1 [0.06; 0.2]	0.06 [0.04; 0.09]	*<*0.01	0.1 [0.07; 0.2]	0.08 [0.04; 0.2]	*<*0.01

Abbreviations: per- and poly-fluoroalkyl substances (PFAS), perfluorononanoic acid (PFNA), perfluorooctanoic acid (PFOA), perfluorohexanesulphonic acid (PFHxS), perfluorooctane sulfonic acid (PFOS), methyl-perfluorooxtane sulfonamide acetic acid (Me-PFOSA-AcOH), perfluorodecanoic acid (PFDeA), perfluoroundecanoic acid (PFUdA), method detection limit (MDL).

1 Multiple MDLs are presented where batches had different LODs with the same lab.

**Table 3:** Estimates of association and 95% confidence intervals (CI) between PFAS* (twofold increase in single natural log-transformed compounds and quantile increase in total mixture) and maternal CES-D scores.

	Overall Sample^1^			Immigrant Women^2^			US Born Women^2^		
	N	Beta (95 % CI)	P-value	N	Beta (95 % CI)	P-value	N	Beta (95 % CI)	P-value
PFNA	474	0.3 (−1.05, 1.7)	0.6	209	0.9 (−1.2, 3.0)	0.4	265	−0.5 (−2.3, 1.3)	0.6
PFOA	475	0.2 (−1.2, 1.7)	0.7	210	0.3 (−2.1, 2.7)	0.8	265	−0.1 (−2.0, 1.8)	0.9
PFHxS	475	−0.09 (−1.4, 1.2)	0.9	210	1.2 (−1, 3.4)	0.3	265	−0.9 (−2.4, 0.6)	0.3
PFOS^⋄^	475	**1.5 (0.2, 2.9)**	**0.03**	210	**2.7 (0.7, 4.7)**	**<0.01**	265	0.3 (−1.5, 2.1)	0.7
Me-PFOSA-AcOH^⋄^	458	**1.4 (0.5, 2.4)**	**<0.01**	200	**2.9 (1.2, 4.7)**	**<0.01**	258	0.6 (−0.4, 1.7)	0.2
PFDeA	444	0.6 (−0.6, 1.8)	0.3	197	0.9 (−1.2, 3.0)	0.4	247	0.3 (−1.1, 1.8)	0.6
PFUdA	457	−0.1 (−1.1, 0.9)	0.8	201	−0.4 (−2.0, 1.2)	0.6	256	0.2 (−1.2, 1.5)	0.8
PFAS Mixture	425	0.5 (−0.3, 1.2)	0.2	187	**1.1 (0.002, 2.3)**	**0.05**	238	−0.09 (−1.0, 0.8)	0.8

Abbreviations: per- and poly-fluoroalkyl substances (PFAS), perfluorononanoic acid (PFNA), perfluorooctanoic acid (PFOA), perfluorohexanesulphonic acid (PFHxS), perfluorooctane sulfonic acid (PFOS), methyl-perfluorooxtane sulfonamide acetic acid (Me-PFOSA-AcOH), perfluorodecanoic acid (PFDeA), perfluoroundecanoic acid (PFUdA).

* Single pollutant models estimated through multiple linear regression; PFAS mixture estimated through quantile g-computation.

1 Models adjusted for maternal age, education, pre-pregnancy body mass index, parity, and immigrant status.

2 Models adjusted for maternal age, education, pre-pregnancy body mass index, and parity.

⋄ Models with interaction term between PFAS exposure and immigrant status in overall sample was significant (p < 0.05).

## Data Availability

Data will be made available on request.

## Data Availability Statement

Per University of California, San Francisco Institutional Review Board approval, the data that support the findings of this study are restricted for transmission to those outside the primary investigative team. Data sharing with investigators outside the team requires IRB approval. Requests may be submitted to the Program on Reproductive Health and the Environment (PRHE).

## References

[R1] Aimuzi R, Luo K, Huang R, Huo X, Nian M, Ouyang F, Du Y, Feng L, Wang W, Zhang J, 2020. Perfluoroalkyl and polyfluroalkyl substances and maternal thyroid hormones in early pregnancy. Environ. Pollut 264, 114557. 32388293 10.1016/j.envpol.2020.114557

[R2] Alegría M, Álvarez K, DiMarzio K, 2017. Immigration and mental health. Current epidemiology reports 2017. 10.1007/s40471-017-0111-2 PMC5966037 29805955

[R3] Blanchard RJ, McKittrick CR, Blanchard DC, 2001. Animal models of social stress: effects on behavior and brain neurochemical systems. Physiol. Behav 73, 261–271. 11438351 10.1016/s0031-9384(01)00449-8

[R4] Budiman Abby, Tamir C, Mora L, Noe-Bustamante L, 2020. Facts on U.S. immigrants, 2018, Statistical portrain of the foreign-born population in the United States

[R5] Calafat AM, Wong L-Y, Kuklenyik Z, Reidy JA, Needham LL, 2007. Polyfluoroalkyl chemicals in the US population: data from the National Health and Nutrition Examination Survey (NHANES) 2003–2004 and comparisons with NHANES 1999–2000. Environ. Health Perspect 115, 1596–1602. 18007991 10.1289/ehp.10598 PMC2072821

[R6] Canady RB, Stommel M, Holzman C, 2009. Measurement properties of the centers for epidemiological studies depression scale (CES-D) in a sample of African American and non-Hispanic White pregnant women. J. Nurs. Meas 17, 91–104. 19711708 10.1891/1061-3749.17.2.91 PMC2997619

[R7] Cao Y, Ng C, 2021. Absorption, distribution, and toxicity of per-and polyfluoroalkyl substances (PFAS) in the brain: a review. Environ. Sci. Process, Impacts10.1039/d1em00228g 34533150

[R8] Dagher RK, Bruckheim HE, Colpe LJ, Edwards E, White DB, 2021. Perinatal depression: Challenges and opportunities. J. Womens Health 30, 154–159. 10.1089/jwh.2020.8862 PMC7891219 33156730

[R9] Dassuncao C, Pickard H, Pfohl M, Tokranov AK, Li M, Mikkelsen B, Slitt A, Sunderland EM, 2019. Phospholipid levels predict the tissue distribution of poly- and perfluoroalkyl substances in a marine mammal. Environ. Sci. Technol. Lett 6, 119–125. 33283018 10.1021/acs.estlett.9b00031 PMC7713714

[R10] De Silva AO, Armitage JM, Bruton TA, Dassuncao C, Heiger-Bernays W, Hu XC, Kärrman A, Kelly B, Ng C, Robuck A, 2021. PFAS exposure pathways for humans and wildlife: a synthesis of current knowledge and key gaps in understanding. Environ. Toxicol. Chem 40, 631–657. 33201517 10.1002/etc.4935 PMC7906948

[R11] Eick SM, Hom Thepaksorn EK, Izano MA, Cushing LJ, Wang Y, Smith SC, Gao S, Park J-S, Padula AM, DeMicco E, 2020. Associations between prenatal maternal exposure to per-and polyfluoroalkyl substances (PFAS) and polybrominated diphenyl ethers (PBDEs) and birth outcomes among pregnant women in San Francisco. Environ. Health 19, 1–12. 32938446 10.1186/s12940-020-00654-2 PMC7495899

[R12] Eick SM, Enright EA, Geiger SD, Dzwilewski KL, DeMicco E, Smith S, Park J-S, Aguiar A, Woodruff TJ, Morello-Frosch R, 2021. Associations of maternal stress, prenatal exposure to per-and polyfluoroalkyl substances (PFAS), and demographic risk factors with birth outcomes and offspring neurodevelopment: an overview of the ECHO. CA. IL prospective birth cohorts. Int. J. Environ. Res. Public Health 18, 742. 33467168 10.3390/ijerph18020742 PMC7830765

[R13] Eick SM, Enright EA, Padula AM, Aung M, Geiger SD, Cushing L, Trowbridge J, Keil AP, Baek HG, Smith S, 2022a. Prenatal PFAS and psychosocial stress exposures in relation to fetal growth in two pregnancy cohorts: Applying environmental mixture methods to chemical and non-chemical stressors. Environ. Int 163, 107238. 35436721 10.1016/j.envint.2022.107238 PMC9202828

[R14] Eick SM, Goin DE, Cushing L, DeMicco E, Smith S, Park J-S, Padula AM, Woodruff TJ, Morello-Frosch R, 2022b. Joint effects of prenatal exposure to per- and poly-fluoroalkyl substances and psychosocial stressors on corticotropin-releasing hormone during pregnancy. J. Expo. Sci. Environ. Epidemiol 32, 27–36. 33824413 10.1038/s41370-021-00322-8 PMC8492777

[R15] Eick SM, Barr DB, Brennan PA, Taibl KR, Tan Y, Robinson M, Kannan K, Panuwet P, Yakimavets V, Ryan PB, 2023. Per-and polyfluoroalkyl substances and psychosocial stressors have a joint effect on adverse pregnancy outcomes in the Atlanta African American Maternal-Child cohort. Sci. Total Environ 857, 159450. 36252672 10.1016/j.scitotenv.2022.159450 PMC9884463

[R16] Environmental Protection Agency, 2000. EPA and 3M ANNOUNCE PHASE OUT OF PFOS

[R17] Fenton SE, Ducatman A, Boobis A, DeWitt JC, Lau C, Ng C, Smith JS, Roberts SM, 2021. Per-and polyfluoroalkyl substance toxicity and human health review: Current state of knowledge and strategies for informing future research. Environ. Toxicol. Chem 40, 606–630. 33017053 10.1002/etc.4890 PMC7906952

[R18] Flores Morales J, Nkimbeng M, 2021. An Exploration of the Relationship Between Diabetes and Depression Among Immigrants in the United States. J. Immigr. Minor. Health 23, 444–451. 33386600 10.1007/s10903-020-01132-0 PMC8076056

[R19] Foguth RM, Hoskins TD, Clark GC, Nelson M, Flynn RW, de Perre C, Hoverman JT, Lee LS, Sepúlveda MS, Cannon JR, 2020. Single and mixture per-and polyfluoroalkyl substances accumulate in developing Northern leopard frog brains and produce complex neurotransmission alterations. Neurotoxicol. Teratol 81, 106907. 32561179 10.1016/j.ntt.2020.106907

[R20] Gee GC, Morey BN, Walsemann KM, Ro A, Takeuchi DT, 2016. Citizenship as privilege and social identity: implications for psychological distress. Am. Behav. Sci 60, 680–704. 37850037 10.1177/0002764216632834 PMC10580256

[R21] Hankerson SH, Moise N, Wilson D, Waller BY, Arnold KT, Duarte C, Lugo-Candelas C, Weissman MM, Wainberg M, Yehuda R, 2022. The intergenerational impact of structural racism and cumulative trauma on depression. Am. J. Psychiatry 179, 434–440. 35599541 10.1176/appi.ajp.21101000 PMC9373857

[R22] Hao R, Qi Y, Hou D-N, Ji Y-Y, Zheng C-Y, Li C-Y, Yung W-H, Lu B, Huang Y, 2017. BDNF val66met polymorphism impairs hippocampal long-term depression by down-regulation of 5-HT3 receptors. Front. Cell. Neurosci 11, 306. 29075179 10.3389/fncel.2017.00306 PMC5643500

[R23] Heller HM, Draisma S, Honig A, 2022. Construct Validity and Responsiveness of Instruments Measuring Depression and Anxiety in Pregnancy: A Comparison of EPDS, HADS-A and CES-D. Int. J. Environ. Res. Public. Health 19, 7563. 35805234 10.3390/ijerph19137563 PMC9266170

[R24] Herzog C, Czéh B, Corbach S, Wuttke W, Schulte-Herbrüggen O, Hellweg R, Flügge G, Fuchs E, 2009. Chronic social instability stress in female rats: a potential animal model for female depression. Neuroscience 159, 982–992. 19356682 10.1016/j.neuroscience.2009.01.059

[R25] Hornung RW, Reed LD, 1990. Estimation of average concentration in the presence of nondetectable values. Appl. Occup. Environ. Hyg 5, 46–51.

[R26] Hurley S, Goldberg D, Wang M, Park J-S, Petreas M, Bernstein L, Anton-Culver H, Nelson DO, Reynolds P, 2018. Time trends in per-and polyfluoroalkyl substances (PFASs) in California women: declining serum levels, 2011–2015. Environ. Sci. Technol 52, 277–287. 29198103 10.1021/acs.est.7b04650

[R27] Johansson N, Fredriksson A, Eriksson P, 2008. Neonatal exposure to perfluorooctane sulfonate (PFOS) and perfluorooctanoic acid (PFOA) causes neurobehavioural defects in adult mice. Neurotoxicology 29, 160–169. 18063051 10.1016/j.neuro.2007.10.008

[R28] Keil AP, Buckley JP, O’Brien KM, Ferguson KK, Zhao S, White AJ, 2020. A quantile-based g-computation approach to addressing the effects of exposure mixtures. Environ. Health Perspect 128, 047004. 32255670 10.1289/EHP5838 PMC7228100

[R29] Liew Z, Goudarzi H, Oulhote Y, 2018. Developmental exposures to perfluoroalkyl substances (PFASs): an update of associated health outcomes. Curr. Environ. Health Rep 5, 1–19. 29556975 10.1007/s40572-018-0173-4 PMC6348874

[R30] Liu M, Nordstrom M, Forand S, Lewis-Michl E, Wattigney WA, Kannan K, Wang W, Irvin-Barnwell E, Hwang S-A, 2022. Assessing exposures to per-and polyfluoroalkyl substances in two populations of Great Lakes Basin fish consumers in Western New York State. Int. J. Hyg. Environ. Health 240, 113902. 34915281 10.1016/j.ijheh.2021.113902

[R31] Loth AK, Drabick DA, Leibenluft E, Hulvershorn LA, 2014. Do childhood externalizing disorders predict adult depression? A meta-analysis. J. Abnorm. Child Psychol 42, 1103–1113. 24652486 10.1007/s10802-014-9867-8 PMC4167173

[R32] Luca DL, Garlow N, Staatz C, Margiotta C, Zivin K, 2019. Societal costs of untreated perinatal mood and anxiety disorders in the United States 10.2105/AJPH.2020.305619 PMC7204436 32298167

[R33] McCormick C, Green M, 2013. From the stressed adolescent to the anxious and depressed adult: investigations in rodent models. Neuroscience 249, 242–257. 22967838 10.1016/j.neuroscience.2012.08.063

[R34] Miranda J, Siddique J, Der-Martirosian C, Belin TR, 2005. Depression among Latina immigrant mothers separated from their children. Psychiatr. Serv 56, 717–720. 15939949 10.1176/appi.ps.56.6.717

[R35] Mutic AD, Barr DB, Hertzberg VS, Brennan PA, Dunlop AL, McCauley LA, 2021. Polybrominated diphenyl ether serum concentrations and depressive symptomatology in pregnant African American women. Int. J. Environ. Res. Public. Health 18, 3614. 33807211 10.3390/ijerph18073614 PMC8037135

[R36] Neisewander J, Peartree N, Pentkowski N, 2012. Emotional valence and context of social influences on drug abuse-related behavior in animal models of social stress and prosocial interaction. Psychopharmacology (Berl.) 224, 33–56. 22955569 10.1007/s00213-012-2853-3 PMC4071609

[R37] O’Mahony JM, Donnelly TT, Raffin Bouchal S, Este D, 2013. Cultural background and socioeconomic influence of immigrant and refugee women coping with postpartum depression. J. Immigr. Minor. Health 15, 300–314. 22711219 10.1007/s10903-012-9663-x

[R38] Oulhote Y, Coull B, Bind M-A, Debes F, Nielsen F, Tamayo I, Weihe P, Grandjean P, 2019. Joint and independent neurotoxic effects of early life exposures to a chemical mixture: A multi-pollutant approach combining ensemble learning and g-computation. Environ. Epidemiol 3. 10.1097/EE9.0000000000000063 PMC7015154 32051926

[R39] Piekarski DJ, Diaz KR, McNerney MW, 2020. Perfluoroalkyl chemicals in neurological health and disease: human concerns and animal models. Neurotoxicology 77, 155–168. 31962063 10.1016/j.neuro.2020.01.001

[R40] Radloff LS, 1977. The CES-D scale: A self-report depression scale for research in the general population. Appl. Psychol. Meas 1, 385–401.

[R41] Rickard BP, Rizvi I, Fenton SE, 2022. Per-and poly-fluoroalkyl substances (PFAS) and female reproductive outcomes: PFAS elimination, endocrine-mediated effects, and disease. Toxicology 465, 153031. 34774661 10.1016/j.tox.2021.153031 PMC8743032

[R42] Slavich GM, Sacher J, 2019. Stress, sex hormones, inflammation, and major depressive disorder: Extending Social Signal Transduction Theory of Depression to account for sex differences in mood disorders. Psychopharmacology (Berl.) 236, 3063–3079. 31359117 10.1007/s00213-019-05326-9 PMC6821593

[R43] Snow G, Melvin GA, Boyle JA, Gibson-Helm M, East CE, McBride J, Gray KM, 2021. Perinatal psychosocial assessment of women of refugee background. Women Birth 34, e302–e308. 32571715 10.1016/j.wombi.2020.05.009

[R44] Sunderland EM, Hu XC, Dassuncao C, Tokranov AK, Wagner CC, Allen JG, 2019. A review of the pathways of human exposure to poly-and perfluoroalkyl substances (PFASs) and present understanding of health effects. J. Expo. Sci. Environ. Epidemiol 29, 131–147. 30470793 10.1038/s41370-018-0094-1 PMC6380916

[R45] Taibl KR, Schantz S, Aung MT, Padula A, Geiger S, Smith S, Park J-S, Milne GL, Robinson JF, Woodruff TJ, 2022. Associations of per-and polyfluoroalkyl substances (PFAS) and their mixture with oxidative stress biomarkers during pregnancy. Environ. Int 169, 107541. 36191484 10.1016/j.envint.2022.107541 PMC9846434

[R46] Troubat R, Barone P, Leman S, Desmidt T, Cressant A, Atanasova B, Brizard B, El Hage W, Surget A, Belzung C, 2021. Neuroinflammation and depression: A review. Eur. J. Neurosci 53, 151–171. 32150310 10.1111/ejn.14720

[R47] Vuong AM, Yolton K, Braun JM, Sjodin A, Calafat AM, Xu Y, Dietrich KN, Lanphear BP, Chen A, 2020. Polybrominated diphenyl ether (PBDE) and poly-and perfluoroalkyl substance (PFAS) exposures during pregnancy and maternal depression. Environ. Int 139, 105694. 32259757 10.1016/j.envint.2020.105694 PMC7275897

[R48] Vuong AM, Yolton K, Xie C, Dietrich KN, Braun JM, Webster GM, Calafat AM, Lanphear BP, Chen A, 2021. Childhood exposure to per-and polyfluoroalkyl substances (PFAS) and neurobehavioral domains in children at age 8 years. Neurotoxicol. Teratol 88, 107022. 34438039 10.1016/j.ntt.2021.107022 PMC8578387

[R49] Wang J, Pan Y, Cui Q, Yao B, Wang J, Dai J, 2018. Penetration of PFASs across the blood cerebrospinal fluid barrier and its determinants in humans. Environ. Sci. Technol 52, 13553–13561. 30362723 10.1021/acs.est.8b04550

[R50] Xie X, Weng X, Liu S, Chen J, Guo X, Gao X, Fei Q, Hao G, Jing C, Feng L, 2021. Perfluoroalkyl and polyfluoroalkyl substance exposure and association with sex hormone concentrations: results from the NHANES 2015–2016. Environ. Sci. Eur 33, 1–12. 10.1186/s12302-021-00508-9 PMC9440377 36061407

[R51] Yu G, Luo F, Nian M, Li S, Liu B, Feng L, Zhang J, 2021. Exposure to Perfluoroalkyl Substances During Pregnancy and Fetal BDNF Level: A Prospective Cohort Study. Front. Endocrinol 376. 10.3389/fendo.2021.653095 PMC8204808 34140927

[R52] Zelkowitz P, Saucier J-F, Wang T, Katofsky L, Valenzuela M, Westreich R, 2008. Stability and change in depressive symptoms from pregnancy to two months postpartum in childbearing immigrant women. Arch. Womens Ment. Health 11, 1–11. 18270652 10.1007/s00737-008-0219-y

