# Supplementary material for: Maternal per- and poly-fluoroalkyl substances exposures associated with higher depressive symptom scores among immigrant women in the Chemicals in Our Bodies cohort in San Francisco
Source: Environ Int. Author manuscript; Available in PMC 2024 Feb 5. (PMC10840585; doi:10.1016/j.envint.2023.107758)
Supplement: 1 [file NIHMS1957999-supplement-1.docx]

**Supplemental Table 1.** Spearman Correlation Coefficients of PFAS compounds (N=521)

|  | | | | |  |  |  |
| --- | --- | --- | --- | --- | --- | --- | --- |
|  | PFNA | PFOA | PFHxS | PFOS | MePFOS | PFDeA | PFUdA |
| PFNA | 1 |  |  |  |  |  |  |
| PFOA | 0.7 | 1 |  |  |  |  |  |
| PFHxS | 0.5 | 0.7 | 1 |  |  |  |  |
| PFOS | 0.7 | 0.6 | 0.6 | 1 |  |  |  |
| MePFOS | 0.2 | 0.2 | 0.2 | 0.3 | 1 |  |  |
| PFDeA | 0.7 | 0.6 | 0.3 | 0.6 | 0.1 | 1 |  |
| PFUdA | 0.6 | 0.4 | 0.3 | 0.5 | 0.1 | 0.8 | 1 |

Abbreviations: per- and poly-fluoroalkyl substances (PFAS), perfluorononanoic acid (PFNA), perfluorooctanoic acid (PFOA), perfluorohexanesulphonic acid (PFHxS), perfluorooctane sulfonic acid (PFOS), methyl-perfluorooxtane sulfonamide acetic acid (Me-PFOSA-AcOH), perfluorodecanoic acid (PFDeA), perfluoroundecanoic acid (PFUdA)

**Supplemental Table 2.** Associations between quartiles of Me-PFOSA-AcOH and CES-D scores to assess potential dose-response relationships.

|  | Overall Sample^1^  (N=458) | | Immigrant Women^2^  (N=200) | | US Born Women^2^  (N=258) | |
| --- | --- | --- | --- | --- | --- | --- |
|  | Beta (95% CI) | P-value | Beta (95% CI) | P-value | Beta (95% CI) | P-value |
| Quartile 1 [0.000029-0.0254] | Ref | - | Ref | - | Ref | - |
| Quartile 2 [0.0254-0.042] | 0.4 (-0.9, 1.7) | 0.55 | 1.5 (-0.5, 3.4) | 0.14 | -0.8 (-2.5, 0.9) | 0.34 |
| Quartile 3 [0.042-0.0692] | **2.3 (1.0, 3.6)** | **0.001** | **3.5 (1.4, 5.7)** | **0.001** | 1.1 (-0.5, 2.7) | 0.18 |
| Quartile 4 [0.0692-1.79] | **1.9 (0.6, 3.3)** | **0.004** | **3.0 (0.8, 5.2)** | **0.008** | 0.9 (-0.8, 2.5) | 0.3 |

**^1^**Models adjusted for maternal age, education, pre-pregnancy body mass index, parity, and immigrant status

^2^Models adjusted for maternal age, education, pre-pregnancy body mass index, and parity

Abbreviations: methyl-perfluorooxtane sulfonamide acetic acid (Me-PFOSA-AcOH)

**Supplemental Table 3.** Relative positive (+) and negative (-) weights^1^ estimated from quantile q-computation for each PFAS

compound within the overall mixture (combined study sample and stratified by immigrant status)

| Combined Study Sample (N=425) | | Immigrant (N=187) | | US Born (N=238) | |
| --- | --- | --- | --- | --- | --- |
| Me-PFOSA-AcOH (+) | 0.43 | Me-PFOSA-AcOH (+) | 0.27 | Me-PFOSA-AcOH (+) | 0.65 |
| PFOS (+) | 0.43 | PFDeA (+) | 0.26 | PFOS (+) | 0.26 |
| PFDeA (+) | 0.14 | PFOS (+) | 0.19 | PFDeA (+) | 0.09 |
| PFUdA (-) | 0.5 | PFHxS (+) | 0.18 | PFNA (-) | 0.48 |
| PFOA (-) | 0.34 | PFNA (+) | 0.1 | PFUdA (-) | 0.23 |
| PFHxS (-) | 0.14 | PFUdA (-) | 0.63 | PFHxS (-) | 0.19 |
| PFNA (-) | 0.008 | PFNA (-) | 0.37 | PFOA (-) | 0.09 |

^1^Positive and negative weights sum to 1

Abbreviations: per- and poly-fluoroalkyl substances (PFAS), perfluorononanoic acid (PFNA), perfluorooctanoic acid (PFOA), perfluorohexanesulphonic acid (PFHxS), perfluorooctane sulfonic acid (PFOS), methyl-perfluorooxtane sulfonamide acetic acid (Me-PFOSA-AcOH), perfluorodecanoic acid (PFDeA), perfluoroundecanoic acid (PFUdA)
